# Protection from Hendra virus infection with Canarypox recombinant vaccine

**DOI:** 10.1038/npjvaccines.2016.3

**Published:** 2016-07-28

**Authors:** Vanessa Guillaume-Vasselin, Laurent Lemaitre, Kévin P Dhondt, Laurence Tedeschi, Amelie Poulard, Catherine Charreyre, Branka Horvat

**Affiliations:** 1 CIRI, International Center for Infectiology Research, Lyon, France; 2 Inserm, U1111, Lyon, France; 3 CNRS, UMR5308, Lyon, France; 4 Université Lyon 1, Lyon, France; 5 Ecole Normale Supérieure de Lyon, Lyon, France; 6 R.E. Merial, SAS, Lyon, France

## Abstract

Hendra virus (HeV) is an emerging zoonotic pathogen, which causes severe respiratory illness and encephalitis in humans and horses. Since its first appearance in 1994, spillovers of HeV from its natural reservoir fruit bats occur on almost an annual basis. The high mortality rate in both humans and horses and the wide-ranging reservoir distribution are making HeV a serious public health problem, especially for people exposed to sick horses. This study has aimed to develop an efficient low-cost HeV vaccine for horses based on Canarypox recombinant vector expressing HeV glycoproteins, attachment glycoprotein (G) and fusion protein (F). This vaccine was used to immunise hamsters and then challenged intraperitoneally with HeV 3 weeks later. The higher tested dose of the vaccine efficiently prevented oropharyngeal virus shedding and protected animals from clinical disease and virus-induced mortality. Vaccine induced generation of seroneutralising antibodies and prevented virus-induced histopathological changes and a production of viral RNA and antigens in animal tissues. Interestingly, some vaccinated animals, including those immunised at a lower dose, were protected in the absence of detectable specific antibodies, suggesting the induction of an efficient virus-specific cellular immunity. Finally, ponies immunised using the same vaccination protocol as hamsters developed strong seroneutralising titres against both HeV and closely related Nipah virus, indicating that this vaccine may have the ability to induce cross-protection against Henipavirus infection. These data suggest that Canarypox-based vectors encoding for HeV glycoproteins present very promising new vaccine candidate to prevent infection and shedding of the highly lethal HeV.

## Introduction

Hendra virus (HeV) along with the closely related Nipah virus (NiV) is a highly pathogenic Henipavirus of the *Paramyxoviridae* family. While HeV appeared in 1994 in Australia in horses and humans,^1^ NiV was first identified in 1998 in Malaysia in pigs and humans.^2^ Both are zoonotic viruses and are able to infect a wide range of mammalian species including pigs, horses, cattle, cats and dogs.^3^ Since their first appearance, numerous outbreaks of both viruses have occurred with evidence of human-to-human transmission and a mortality rate that can approach 75% for NiV.^4^ Between 1994 and 2010 there were a total of 14 HeV outbreaks. In 2011, within a 3-month period, there were 18 unprecedented observations of emergences of HeV in horses over an expanded geographic range.^5^ In 2012, eight outbreaks occurred, emphasising that HeV is an unmanaged emerging disease. Flying foxes of the genus *Pteropus* are considered to be the natural reservoir for Henipaviruses, and their geographic distribution includes all regions where HeV and NiV outbreaks have occurred. Transmission and spillover infection is thought to occur through food contaminations or direct contact with secretions from infected animals.^6,7^ Horses become infected when the HeV spills over from *Pteropus* flying foxes and infection could be transmitted to humans following the exposure to the secretions of infected horses. HeV has low infectivity in horses and humans but a high mortality rate in both species (75% and 57% respectively).^8^ Consequently, HeV is considered at high economical risk for horse breeding and at high occupational risk regarding the people coming into contact with infected horses.^9^

Horse-to-human transmission is currently confined to people exposed to sick horses, thus rather favoring the vaccination approach in horses. The first evidence of antibody (Ab)-mediated protection against HeV infection was shown using monoclonal antibodies specific for NiV glycoproteins in hamsters.^10^ The human monoclonal antibody m120.4, specific for HeV glycoprotein G, with the capacity to neutralise both HeV and NiV infection,^11^ was shown to protect African green monkeys against HeV infection.^12^ Though, the most direct strategy for reducing the risk posed by HeV-infected horses to both horse industry and human health is employment of an approach that would lead to the control of infection in horses. The development of efficient vaccine approach for Henipavirus infection has focused on the use of Henipavirus glycoprotein (G) and/or fusion protein (F) as immunogens in various platforms, including DNA vaccines, subunit vaccines, non-replicating as well as replicating vectors.^13^ A recombinant HeV G glycoprotein-based vaccine was shown to protect ferrets,^14^ horses^15^ and nonhuman primates^16^ against lethal HeV challenge, and this vaccine has recently been commercialised for horses in Australia. Furthermore, recombinant vectors, derived from Vaccinia virus or Canarypox virus, were shown to induce a humoral response against the NiV G and/or F proteins, which could protect hamsters^17^ and pigs,^18^ respectively, against a challenge with wild-type NiV. Canarypox vector infection leads to the abundant production of viral proteins in different cell types, but replication is blocked prior to the level of DNA synthesis, thus leading to the abortive infection in mammalian cells and eliminating the safety concerns that exist for vaccinia virus vectors.^19^ Several Canarypox vaccines against veterinarily important pathogens, including canine distemper virus, rabies and influenza, are commercially available.^20^ Finally, Canarypox-based human HIV-1 vaccine trial has demonstrated the efficacy against the HIV-1 acquisition.^21^

We describe in this manuscript the development and evaluation of Canarypox-vectored (ALVAC) vaccines expressing HeV glycoproteins G and F for horses. We have initially assessed the protective efficacy of the new vaccine in hamsters and analysed virus replication and shedding. We further characterised the humoral immune response to vaccination in hamsters using enzyme-linked immunosorbent assay (ELISA) and neutralisation assays. Finally, the Ab response to vaccination in ponies was investigated. Altogether, the obtained results suggest that this ALVAC vaccine could confer protection against Hendra infection and thus present a good candidate for a new efficient vaccination of horses, with the potential for breaking the chain of HeV transmission from bats to horses and then to humans, thereby protecting both horse and human health.

## Results

### Expression of HeV glycoproteins G and F by Canarypox-based vaccines

**Figure 1:**
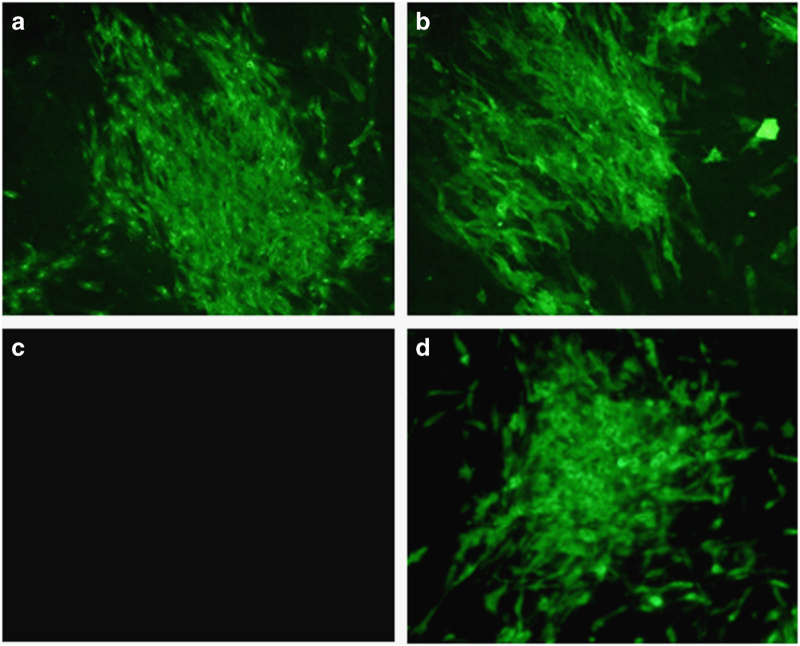
Expression of HeV G and F in infected primary CECs. CECs were infected with ALVAC HeV.G and ALVAC-HeV.F at 7 log_10_ CCID_50_/ml, (**a**, **b**) or with empty Canarypox vector, vCP (**d**), or left uninfected (**c**). Cells were stained 48 h later with polyclonal murine anti-HeV F Ab (**a**, **c**), polyclonal murine anti-HeV G Ab (**b**, **c**) or anti-vCP mAb (**d**), followed by rabbit anti-mouse-FITC. Images from representative wells are presented. Ab, antibody; CEC, chicken embryonic cell; FITC, fluorescein isothiocyanate; HeV, Hendra virus; mAb, monoclonal antibody.

The expression of HeV G and F proteins by ALVAC-HeV.G and ALVAC-HeV.F vaccines was analysed in primary chicken embryonic cells. Specific staining of both HeV G and HeV F was revealed by immunofluorescence (Figure 1), suggesting that Canarypox (ALVAC)-based vectors allow good expression of HeV glycoproteins.

### Vaccination by ALVAC HeV G and F provides efficient protection from lethal challenge with HeV in hamsters

**Figure 2:**
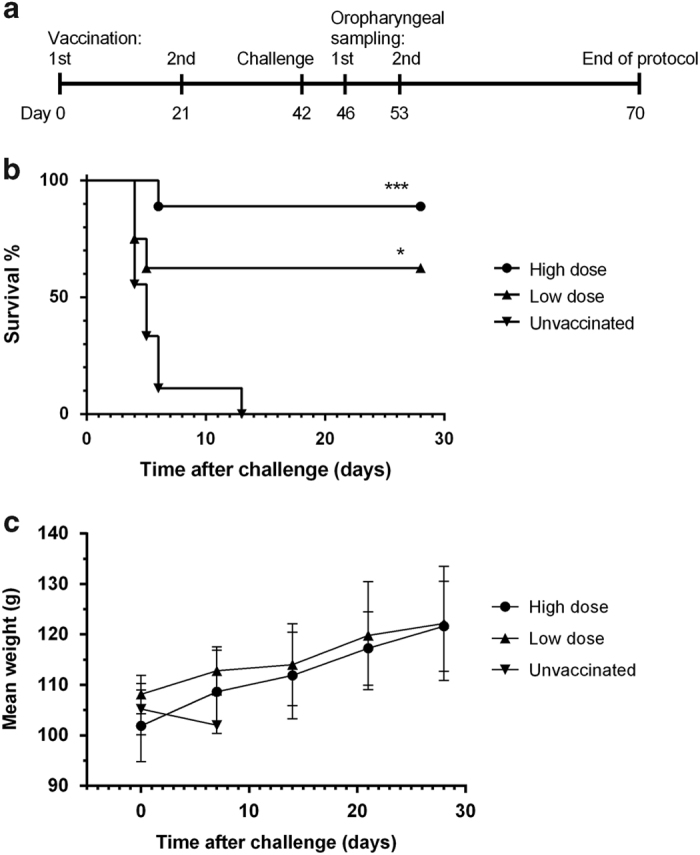
Protection of hamsters from the lethal HeV challenge by vaccination with ALVAC expressing HeV G and F glycoproteins. (**a**) Flow chart of the study. Hamsters were vaccinated twice at 21 days interval with different doses of both vaccines (ALVAC-HeV.G and ALVAC-NiV.F): group ‘high dose’ received 7.4 log_10_ CCID_50_ of ALVAC vaccine (*n*=9) and group ‘low dose’ received 5.4 log_10_ CCID_50_ of ALVAC vaccine (*n*=8). Control group (*n*=9) was left unvaccinated. All groups were challenged with HeV (10^4^ plaque-forming units (PFU), i.p.) 21 days after the last immunisation. Four and eleven days after the challenge oropharyngeal samplings were performed on all surviving animals. (**b**) Animals were examined daily for 28 days after the challenge. The occurrence of the first signs of neurological impairment has resulted in death and necropsy of animals. All surviving animals were killed and necropsied at the end of the protocol (day 70). Results are expressed as the percentage of animals that survived over time. ***P value<0.0001 for 7.4 log_10_ CCID_50_/ml versus unvaccinated; *P value=0.016 for 5.4 log_10_ CCID_50_/ml versus unvaccinated, Mantel–Cox test, with similar variance between the groupes. (**c**) Weight curves of infected hamsters presented as average±s.d. at indicated time points.

Hamsters have previously been shown to be a suitable animal model to test innovative therapeutic strategies against HeV infection in BSL-4 conditions.^10^ To evaluate the protection efficacy of the ALVAC-HeV.G and ALVAC-HeV.F vaccines against lethal HeV infection, the vaccine was inoculated subcutaneously into hamsters on days 0 and 21 (Figure 2a). As previous study using ALVAC-expressing individual NiV G and F glycoproteins in pigs demonstrated that combination of both is more efficient than the vaccines administrated separately,^18^ in this study only combination of HeV G- and HeV F-expressing vaccines in two different doses was evaluated. High and low doses of vaccine containing respectively 7.4 log_10_ CCID_50_ and 5.4 log_10_ CCID_50_ of both vaccines were tested. Vaccinated and control hamsters were infected by the intraperitoneal route with 1,000 LD_50_ of HeV, based on our previous results testing HeV infection in hamsters.^10^

Animals in the unvaccinated group developed clinical signs of disease between 4 and 13 days after infection resulting in respiratory distress with varying degrees of neurologic dysfunction. In contrast, most vaccinated animals did not show clinical signs: in eight out of nine hamsters (89%) vaccinated with the high dose of vaccine, complete protection against the lethal infection was observed, with no significant weight loss or temperature variation (data not shown). In addition, five out of eight hamsters (63%) vaccinated with the low dose were also protected from clinical disease (Figure 2b). Finally, in contrast to unvaccinated animals, vaccinated hamsters did not show any weight loss during 1 month after the challenge (Figure 2c).

### Induction of anti-HeV antibodies by ALVAC HeV F and G vaccination in hamsters

**Figure 3:**
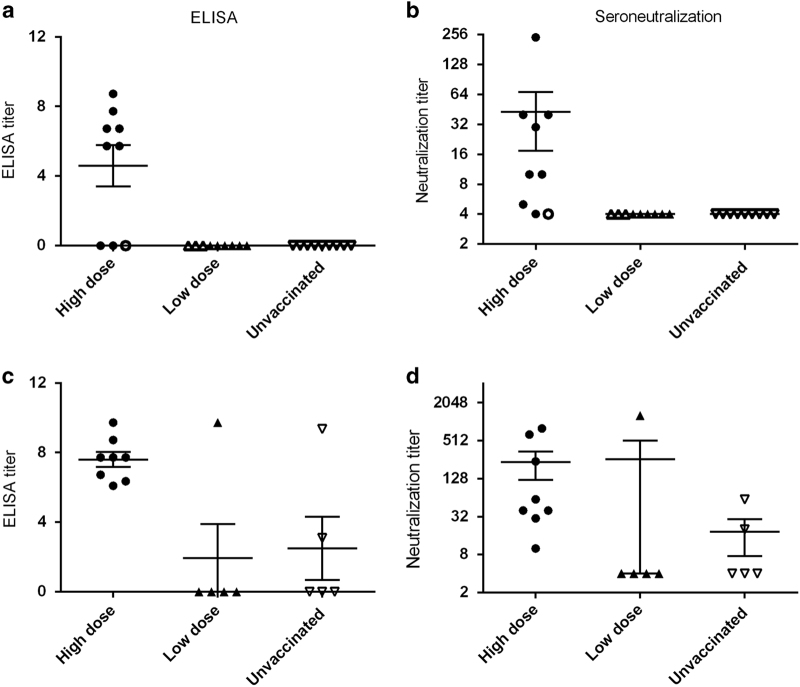
HeV-specific humoral response after vaccination (day 38; **a**, **b**) and after vaccination and challenge (at the end of the protocol, day 70, or at necropsy; **c**, **d**). Levels of HeV-specific Abs were measured by ELISA and results were log-transformed to attenuate outlier effect on graphical presentation (**a**, **c**). For HeV seroneutralisation assay, serum dilutions, starting from 1:10, were assayed in duplicates for the presence of virus-neutralising antibodies and results are presented in Log2 scale, to reflect the half dilution applied in the test. (**b**, **d**) Horizontal lines correspond to the average titre±s.d. Filled symbols represent hamsters without clinical symptoms by the end of the protocol, whereas empty symbols indicate animals that developed symptoms during the protocol. Ab, antibody; ELISA, enzyme-linked immunosorbent assay; HeV, Hendra virus.

Humoral immune responses after vaccination with ALVAC-HeV.G and ALVAC-HeV.F were assessed by measuring HeV-specific antibodies in sera of vaccinated hamsters. Sera were taken on day 38 before the challenge with HeV. As shown in Figure 3, anti-HeV antibodies were induced in six out of nine (67%) of the hamsters vaccinated with the high dose of vaccine, but in none of the hamsters vaccinated with low dose of vaccine. All hamsters that developed anti-HeV antibodies detected by ELISA had also neutralising antibodies. Ab titres (ELISA and neutralising antibodies) were also measured after the challenge on the day of killing. Hamsters that had developed anti-HeV antibodies and neutralising antibodies after the vaccination showed an increase in their Ab titre after the challenge. Some hamsters (three out of nine) that had no detectable antibodies before the challenge developed neutralising antibodies after the challenge. Surprisingly, four protected hamsters in ‘low-dose’ group did not develop anti-HeV antibodies, detectable neither by ELISA nor by seroneutralisation, tested after the vaccination or after challenge, suggesting that the ALVAC vaccine induced a protective cell-mediated immune response, allowing the survival of animals to the lethal HeV challenge.

### Vaccination reduced the nasal and pharyngeal shedding of HeV in hamsters

**Figure 4:**
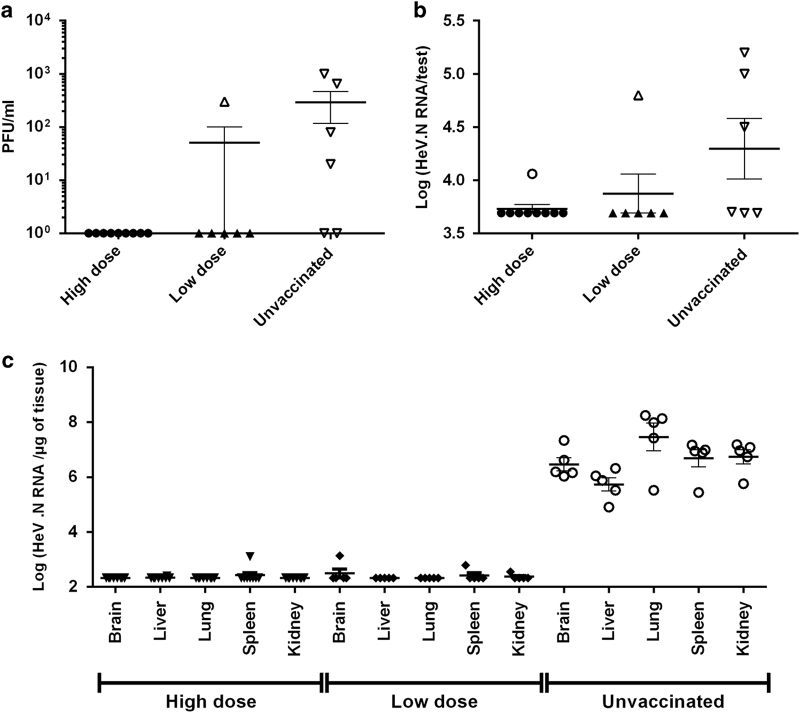
Vaccination reduces oropharingeal shedding and viral load in HeV-infected hamsters. Oropharyngeal swabs were collected on day 4 after the challenge, and the viral load was determined in the swabs by titration as PFU/ml (**a**) and as HeV.N RNA copies, detected using RT-qPCR (**b**). The detection threshold of titration assay was 10 PFU/ml, and the detection threshold of the RT-qPCR assay was 3.69 log (HeV.N RNA copies/test). (**c**) Viral load in organs of infected hamsters was detected by measuring viral HeV.N RNA copies per μg of the brain, liver, lung, spleen and kidney, obtained after necropsy, using RT-qPCR. Horizontal lines correspond to the average values±s.d. Filled symbols represent hamsters without clinical symptoms by the end of the protocol, whereas empty symbols indicate animals that developed symptoms during the protocol.

HeV has been previously isolated from horse urine, saliva, nasal and oropharyngeal secretions, and epidemiological data have indicated that direct contact with these secretion results in risk of infection and may be the most efficient route of HeV transmission.^7^ To analyse the effect of vaccination on virus shedding, oropharyngeal swabs were collected on days 4 and 11 after the challenge and analysed viral load was determined by titration and reverse transcriptase quantitative PCR (RT-qPCR) as HeV-N RNA copy numbers. As shown in Figure 4a, in protected vaccinated hamsters viral RNA was not found in any of oropharyngeal swabs. On day 4 after the challenge, HeV was detected in one out of nine oropharyngeal swabs from hamsters vaccinated with the high dose, only by RT-PCR, in the animal that succumbed to the infection. In addition, HeV was found in one out of six (16.6%) hamsters vaccinated with a low vaccine dose. In contrast, in four out of six (66.6%) unvaccinated hamsters’ oropharyngeal swabs were found positive for HeV (Figure 4a,b). On day 11, HeV was not detected either with RT-qPCR or with titration in any of oropharyngeal swabs from vaccinated animals, but was found in one unvaccinated hamster, which was still alive on day 11 (data not shown).

### Vaccination reduced viral loads in infected hamsters

To determine the effect of vaccination on virus replication, different organs were collected either at the end of the protocol or when hamsters were found sick and killed during the protocol and isolated RNA was analysed using RT-qPCR. Whereas in unvaccinated animals viral RNA was easily detected at high level in all organ tissue samples, expression of the HeV N was found only at the background levels in tissues of analysed vaccinated animals (Figure 4c).

### Immunohistopathology in infected animals

**Figure 5:**
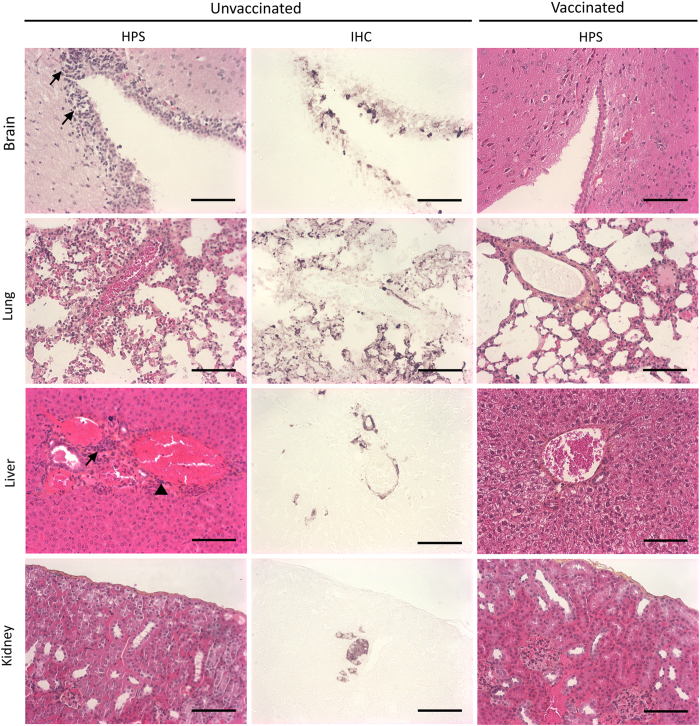
Vaccination reduces HeV pathology in infected hamsters. Hamsters were killed after appearance of symptoms of infection or at the end of protocol. Brain, lung, kidney and liver sections were stained with HPS for histopathological analysis, and immunohistochemistry was performed targeting HeV N protein for virus replication and localisation of viral antigens. Positive staining was found in all analysed organs of unvaccinated hamsters, whereas all vaccinated animals remained negative (data not shown). Histopathology revealed strong inflammation in the brain, lungs and liver with vasculitis, massive leukocyte infiltration (arrows) and syncitia formation (arrowhead) in unvaccinated animals, whereas vaccinated animals remained normal with a mild inflammation in lungs. Bar = 100 μ. HPS, haematoxylin/phloxine B/saffron; IHC, immunohistochemistry.

Upon necropsy, HeV-infected unvaccinated hamsters exhibited congestion with scattered small haemorrhagic lesions in the brain and lungs, whereas organs from vaccinated animals did not present any visible lesions. Paraformaldehyde-fixed and paraffin-embedded tissues from several organs of both vaccinated and non-vaccinated hamsters were analysed for histopathological changes and presence of viral antigens (Figure 5). In unvaccinated animals, histopathology of lungs revealed inflammation with oedema, focal necrotising alveolitis and vasculitis. Focal necrosis and petechial haemorrhages were observed in the liver and kidney. Viral antigens were detected in the brain, kidney, liver and lungs (Figure 5, immunohistochemistry). In contrast, vaccinated animals presented normal histopathology in all analysed organs and viral antigens were not detected.

### Vaccination of ponies by ALVAC HeV G and F induces a high level of anti-HeV-neutralising antibodies

**Figure 6:**
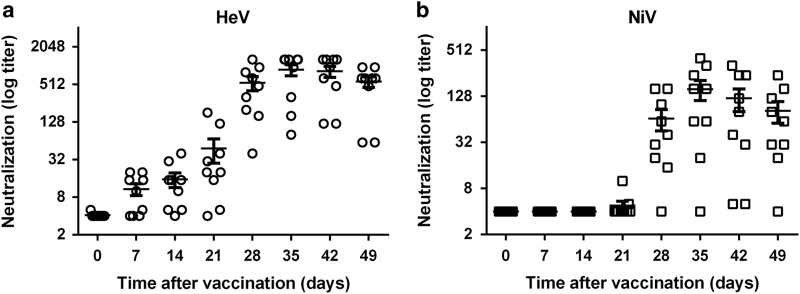
Vaccination induces strong Henipavirus-specific humoral response in ponies. Nine ponies were vaccinated twice (days 0 and 21) with ALVAC-HeV.G and ALVAC-HeV.F and sera were collected at the indicated time points and tested for the presence of HeV-neutralising Abs (**a**) and NiV-neutralising antibodies (**b**). The detection threshold was 2.32 log for each neutralisation assay and the mean neutralisation titre±s.d. is presented for each analysed time point as a horizontal line. Results are presented in log2 scale to reflect the half dilution of sera used in the assay.

To evaluate the efficacy of the vaccine when used in equines, nine ponies were vaccinated twice, 21 days apart (target minimum protective doses). Owing to the limited availability of ponies, only an intermediary dose of vaccine (6.0 log_10_CCID_50_) was used for the immunisation. Vaccination induced a high level of neutralising anti-HeV antibodies starting at 28 days after vaccination (Figure 6a), with titres of at least 32, which was shown in previous studies to be protective against development of HeV clinical disease in horses.^15^ Interestingly, antibodies induced by the ALVAC HeV vaccination cross-neutralised with the closely related NiV, although at four times lower titre, suggesting that ALVAC may be used to protect equines against NiV infection as well (Figure 6b). While seven out of nine vaccinated ponies developed HeV-specific neutralising antibodies already after the first dose of vaccine, both HeV- and NiV-seroneutralising antibodies were highly increased after the second vaccine boost on day 21. This secondary response reached the highest level 1 week after secondary immunisation and remained at the plateau by the end of the experiment.

## Discussion

Henipaviruses attract particular attention among members of the *Paramyxovirus* family, as they possess a high zoonotic potential, associated with one of the highest mortality rates observed in infectious diseases. Wide distribution of the Henipavirus natural host (fruit bats) raises the risk of potential pandaemics caused by this virus in the future^22^ and urges the better understanding of its pathogenesis and the development of efficient antiviral approaches.^23^ In this study we evaluated two different doses of ALVAC vaccines expressing HeV glycoproteins for their capacity to induce protective immune response in hamsters. The higher tested dose (7.4 log_10_ CCID_50_) efficiently protected animals from the clinical disease and death, inducing the humoral immune responses and inhibiting the viral oropharingeal shedding. In addition, neither viral RNA nor viral antigens and histopathological changes were detected in organs of vaccinated animals, in contrast to unvaccinated hamsters, in which these changes were frequently observed. Highly austere experimental conditions used for HeV challenge (intraperitoneal infection with 1,000 LD_50_ of the virus) circumventing mucosal immunity effective during the natural HeV infection in animals thus largely explain apparent lack of complete vaccine protection, particularly at the lower vaccine dose. Furthermore, these results suggest the importance of establishing the minimum protective vaccine dose for the future clinical trials.

The seroneutralisation titres generated in vaccinated ponies were higher than 32, shown in previous studies to be protective against development of HeV clinical disease in horses,^15^ strongly suggesting that the ALVAC vaccine may protect against HeV challenge in horses. The Ab responses remained at the plateau level after the second challenge during the whole experiment, suggesting the development of the memory response in ponies. However, in contrast to the single-dose vaccination against HeV, using live attenuated or replication-defective vesicular stomatitis virus vaccine,^24–27^ ALVAC vaccine required two doses for the effective generation of Ab responses and protection. Importantly, the ALVAC vaccine induced cross-neutralising to NiV, indicating its protective potential against this infection as well. This cross-neutralisation is in accord to previous reports, showing that HeV and NiV glycoproteins share high sequence homology^28^ and recombinant HeV G glycoprotein-based subunit vaccine protects ferrets, cats and monkeys against both HeV and NiV challenge.^14,27,29–32^ A recent outbreak of NiV in the Philippines in 2014, associated with NiV infection of horses,^33^ underlines the importance of the risk for new upcoming spillovers of Henipavirus and possible necessity to protect horses from Nipah infection in addition to HeV.

In contrast to horses, although all animals developed seroneutralising antibodies, humoral response in hamsters was less evident, probably reflecting the differences in immune response between two animal species. Intriguingly, whereas none of the hamsters immunised with low dose of ALVAC vaccine showed any detectable HeV-specific antibodies in the serum, five out of eight hamsters (63%) were protected against the development of the clinical disease and lethality. Although we could not exclude that some low level of neutralising antibodies may have not been detected in the applied assays, this finding strongly suggests that ALVAC HeV vaccine-induced protection might be related to the activation of the cellular immune response, the induction of which requires lower antigen dose compared with the induction of humoral response. Although the induction of cellular immunity against HeV by ALVAC remains to be additionally characterised, this vaccine was shown to induce both humoral and cellular immunity against different viral pathogens, including related NiV in pigs^18^ and equine influenza virus in horses.^34^ Interestingly, in the feline leukaemia virus model in cats, ALVAC vaccination was shown to be protective in the absence of virus-neutralising antibodies,^35^ whereas the correlation between cell-mediated immunity and protection following the vacciantion was observed.^36^ Moreover, the role of adaptive cellular immunity was suggested in the protection from HeV infection, using recombinant adeno-associated virus vectors^37^ and from NiV infection using recombinant vesicular stomatitis virus.^24^ In addition, the vaccination using Newcastle disease virus-vectored NiV vaccine was shown to induce both specific Ab and interferon γ-producing T-cell responses in mice.^38^ Furthermore, the induction of an adaptive T-helper 1 immune response against NiV G was suggested to occur following the injection of ALVAC NiV G in pigs.^18^ The possibility that ALVAC could induce both humoral and cellular immunity against HeV may have particular advantages to the existing subunit vaccine in horses, which mainly targets the Ab induction.^15^

Altogether, our results have demonstrated that ALVAC vaccine expressing HeV glycoproteins presents an attractive and efficient alternative for the HeV vaccination. Implementation of HeV vaccination of horses at risk in Australia integrates into One Health approach bridging veterinary and medical sciences and could lead to the containment of the future Hendra outbreaks and contribute to both animal and human health.

## Materials and methods

### Cells and viruses

African green monkey fibroblasts (Vero) and Baby Hamster Kidney fibroblasts (BHK 21; American Type Culture Collection) were cultured in Dulbecco’s modified Eagle’s medium (DMEM, Gibco, Invitrogen, Cergy Pontoise, France) supplemented with 10% fetal calf serum (FCS, Biowest, Nuaille, France) and 1% penicillin–streptomycin (5,000 U/ml, Gibco, 15140). Primary CECs were obtained from seven- to nine-day-old embryos and cultured in DMEM (Gibco, Invitrogen) supplemented with 6% FBS, 2 mM L-Glutamine, 10 mM HEPES buffer, 10% tryptose phospahte broth, 2% heat-inactivated chicken serum, 5×10^−5^ mol/l 2-mercapthoethanol and 50 μg/ml gentamycin and incubated at 37 °C with 5% CO_2_. All cells were tested as mycoplasma-free. NiV (isolate UMMC1; GenBank accession number AY029767) and HeV (Hendra virus/Australia/Horse/1994/Hendra), kindly provided by Porton Down Laboratory (Porton Down, UK), were prepared and propagated at the INSERM Jean Mérieux biosafety level 4 (BSL4) laboratory (Lyon, France) by infecting Vero cells as previously described.^17^

### Vaccine preparation

Canarypox-based recombinant virus vectors, ALVAC-HeV.G and ALVAC-HeV.F, carrying the HeV glycoproteins G and F, respectively, were used in the study as a freeze-dried vaccine (Merial batch 27423B051 in hamsters and 27423B041 in ponies). Vaccine titres were determined on CEC cells. Before immunisation, vaccines were reconstituted in Carbopol adjuvant (Lubrizol).

### Hamster immunisation and challenge

All animals were handled in strict accordance with good animal practice, as defined by the French national charter on the ethics of animal experimentation, and experiments were approved by Regional Ethical Committee CECCAPP. Groups of 4-week-old female hamsters (Janvier, France) contained nine animals each, with size of the group chosen to ensure the adequate power to obtain statistically relevant data and comply to the number of animals possible to analyse in the BSL4 conditions. Hamsters were identified with microchip PLEX IPTT300 introduced subcutaneously in flank and were immunised by subcutaneous route with vaccine preparations on days 0 and 21, without randomisation and blinding. Each animal received the same 1-ml dose of vaccine for both prime and boost inoculations, containing either 7.4 log_10_ CCID_50_ of ALVAC-HeV.G and ALVAC-HeV.F (‘high-dose’ group) or 5.4 log_10_ CCID_50_ of ALVAC-HeV.G and ALVAC-HeV.F (‘low-dose’ group). Twenty-one days after the boost (D42), hamsters were infected intraperitonealy with a lethal dose of HeV (10^4^ PFU, corresponding to the 1,000 LD_50_). Animals were housed in ventilated cages in the BSL4 laboratory, observed daily for the appearance of clinical signs (body weight, dyspnoea, tremor and limb paralysis) and immediately killed at the onset of symptoms of clinical disease. Animals that survived infection were killed 28 days after the challenge. Necropsies were performed and sera were collected whenever possible.

### Immunisation of ponies

Nine conventional ponies (seronegative against HeV), male and female mice, 22–36-month old, were vaccinated twice at a 21-day interval (D0 and D21), by intramuscular route with 1 ml of ALVAC-HeV.G and ALVAC-HeV.F vaccine (6.0 log_10_CCID_50_/ml). None of the ponies demonstrated any side effects as a result of vaccination. All animals were handled in strict accordance with good animal practice, as defined by the French national charter on the ethics of animal experimentation, and experiments were approved by the Regional Ethical Committee.

### Sample collection

Sera were systematically collected from hamsters 4 days before the HeV challenge (D38, retro-orbital route) and at the end of the protocol (D70, intracardiac route). Sera were also collected from animals that were killed as a result of onset of symptoms of clinical disease.

Oropharyngeal swabs were collected from hamsters 4 and 11 days after infection (D46 and D53) using cotton swab (Deltalab, Barcelona, Spain). Immediately following sampling, swabs were placed in 0.35 ml of DMEM supplemented with 2% FCS. A volume of 0.11 ml was used in PFU titration assay (PFU/ml), 0.14 ml were used in the RT-qPCR assay and 0.1 ml were conserved in the BSL4 laboratory.

Brain, lung, heart, spleen and kidney tissue samples were taken at the end of the protocol or as soon as possible following killing of symptomatic animals. A first portion of each organ was frozen at −80 °C in the BSL4 laboratory before RNA extraction. A second portion was fixed in 4% formaldehyde for a duration of 14 days and then processed for histopathological analysis.

In ponies, blood samples were taken from the jugular vein of each animal on D0 (prior to first-dose vaccination), D7, D14, D21 (prior to second-dose vaccination), D28, D35, D42 and D49, processed into sera and stored frozen at −20 °C.

### Enzyme-linked immunosorbent assay

Sera were tested individually by ELISA for the presence of anti-HeV antibodies either for anti-N or anti-whole HeV antigen. Crude extracts of HeV antigens were prepared from BHK 21 cells infected at a multiplicity of infection of 0.01 PFU/cell for 24 h. The cells were washed with phosphate-buffered saline (PBS) and lysed in PBS containing 1% Triton X-100 (10^7^ cells/ml) at +4 °C for 10 min. The cell lysate was sonicated twice for 30 s each to full-cell destruction and centrifuged at 5,000 r.p.m. at +4 °C for 10 min. The supernatant was frozen at −80 °C. Non-infected Vero cells were similarly treated to prepare control antigen. NiV N protein was produced in a baculovirus system as previously described^39^ and used in comparison assays. Microtitre plates (96-well plates; Dominic Dutscher) were coated with 200 μl/well of HeV antigen or with 200 μl of N overnight at 4 °C. Wells were blocked by incubation with 3% skim milk in PBS containing 0.05% Tween 20 for 30 min at 37 °C (300 μl per well). Between each step, the wells were washed three times with PBS containing 0.05% Tween 20. Serial dilutions (1/20 and then 1/3 serial dilution until 1/43,740) of hamster sera were individually added and incubated overnight at +4 °C. Horseradish peroxidase (HRP)-conjugated goat anti-hamster IgG (H+L; Southern Biotech, Birmingham, AL, USA; 1/4,000) or HRP rabbit anti-horse IgG (whole molecule) peroxidase (Sigma-Aldrich, St Quentin Fallavier, France; 1/10,000) were used as secondary antibodies for 1 h at 37 °C. Plates were incubated with *O*-phenylenediamine dihydrochloride (Sigma-Aldrich) peroxidase substrate and reaction was stopped with 12% H_2_SO_4_. The optical density was read at 492 and 665 nm. Each result obtained at 492 nm was deducted first by the result obtained at 665 nm and second by the background (PBS) plus three times the s.d. of the background. A serum sample is considered positive when its value is greater than three times the average value obtained with negative sera at the same dilution.

### Seroneutralisation assays

Neutralising Ab titres were determined in Vero cells as described previously.^40^ Briefly, ponies’ and hamsters’ serum dilutions (1:2) in DMEM containing 2% FCS starting with 1:10 were tested in duplicates. In these dilutions, sera from control animals, non-infected and nonimmunised, used in each test, resulted in absence of any detectable seroneutralisaiton, thus providing opitmal conditions to the assay. They were then were mixed with 25 PFU of HeV or NiV in 96-well plates and incubated for 1 h at 37 °C, and then 20,000 Vero cells were added. The plates were read after 5 days following the crystal violet staining, and relative neutralising titres were defined as the reciprocal dilution of sera samples that completely inhibited the cytopathic effect of either HeV or NiV.

### qRT-PCR analysis

After a first step using RLT buffer (Qiagen, Venlo, Netherland) supplemented with 0.1% beta-mercaptoethanol, RNA was purified from tissues in accordance with the manufacturer’s instructions (Kit NucleoSpin RNA Macherey Nagel). Purified RNA was then treated with Turbo DNaseI (Ambion, Life Technologies, Delhi, India) and subjected to reverse transcription using the iScript cDNA Synthesis Kit (Bio-Rad, Marnes La Coguette, France).

RNA from oropharyngeal swabs was extracted and purified using the QIAamp viral RNA Mini Spin kit (Qiagen). The qRT-PCR reaction was conducted on 10 ng of complementary DNA, using the PlatinumSYBRGreen qPCR SuperMix-UDG with ROX (Invitrogen) was run on the Step One Plus Real Time PCR System (Applied Biosystems, Waltham, MA, USA). Sequences of primers that target HeV NP gene and GAPDH gene were as previously described.^37^ All samples were run in duplicate, and results were analysed using the ABI StepOne software v2.1 (Applied Biosystems).

### Virus titration

The viral supernatant from each oropharyngeal swab was titrated in six-well plates by incubating either 100 or 10 μl of the swab extract with 10^6^ Vero cells, respectively, in 500 or 590 μl of DMEM containing 2% FCS for 1 h at 37 °C. Wells were then washed twice with DMEM 2% and recovered by 2 ml DMEM containing 5% FCS and 1.6% carboxymethylcellulose (Sigma). The plates were incubated for 5 days at 37 °C, and wells were washed with PBS (pH 7.4), fixed with 4% formalin for 20 min, washed and stained with crystal violet.

### Histopathology, immunohistochemistry and immunofluorescence

Histology and immunohistochemistry were performed as previously described.^40^ Briefly, tissues were embedded in paraffin wax, sectioned at 3–4 μm and slides were rapidly coloured by Phloxin B-0.1% (Sigma-Aldrich) and Saffron, and then washed. Modified Harris haematoxylin (Sigma-Aldrich) 1:3 in PBS was used for counterstaining. Coloured slides were dehydrated through graded alcohols and xylene. Sections were mounted with DPX (distyrene, plasticiser, xylene) mounting medium and coverslipped. For immunohistochemistry study a primary rabbit anti-NiV N purified Ab (ValBex, Villeurbanne, France)^40^ was applied diluted at 1:1,000 in PBS–bovine serum albumin 1%, overnight at 4 °C. The ImmPRESS anti-rabbit Ig (Eurobio, Les Ulis, France) and the Substrat Peroxydase ImmPACT (Eurobio) were then used. Further steps were performed as described.^40^ For the immunofluorescence assay, cells were attached on six-well plate glass coverslips and left at 37 °C, 5% CO_2_ for 24 h; medium was then removed and cells were infected with ALVAC for 1 h. Following 48 h of infection, cells were fixed using 4% parafolmaldehyde in PBS (1 ml/well, 10 min) at room temperature, followed by wash with PBS. Cells were then permeabilised using Triton X-100 (0.1% in PBS, 3–5 min, at RT) and washed again. Blocking was performed using 3% bovine serum albumin in PBS, 30 min at RT. Cells were then incubated with either primary mouse polyclonal anti-HeV F or anti-HeV G Ab, or anti-vCP monoclonal antibody (Merial) for 1–2 h at 37°. After three washes, cells were incubated with secondary rabbit anti-mouse fluorescein isothiocyanate conjugate (Sigma) for 30 min at 37 °C. After additional three washes in PBS, and one wash in water, slides were mounted and analysed using fluorescence microscope.

### Statistical analyses

Data are expressed as mean±s.e.m. Statistical analyses were performed using the two-tailed Student’s *t*-test, one-way analysis of variance or the Mantel–Cox test with the GraphPad software (La Jolla, CA, USA). Differences were considered statistically significant at *P*<0.05. The normal distribution was assessed by D’Agostino-Pearson omnibus normality test.
